# Properties of Retinal Precursor Cells Grown on Vertically Aligned Multiwalled Carbon Nanotubes Generated for the Modification of Retinal Implant-Embedded Microelectrode Arrays

**DOI:** 10.1155/2016/2371021

**Published:** 2016-04-21

**Authors:** Sandra Johnen, Frank Meißner, Mario Krug, Thomas Baltz, Ingolf Endler, Wilfried Mokwa, Peter Walter

**Affiliations:** ^1^Department of Ophthalmology, University Hospital RWTH Aachen, Pauwelsstraße 30, 52074 Aachen, Germany; ^2^Fraunhofer Institute for Ceramic Technologies and Systems, Winterbergstraße 28, 01277 Dresden, Germany; ^3^Institute for Materials in Electrical Engineering 1, RWTH Aachen University, Sommerfeldstraße 24, 52074 Aachen, Germany

## Abstract

*Background.* To analyze the biocompatibility of vertically aligned multiwalled carbon nanotubes (MWCNT), used as nanomodification to optimize the properties of prostheses-embedded microelectrodes that induce electrical stimulation of surviving retinal cells.* Methods.* MWCNT were synthesized on silicon wafers. Their growth was achieved by iron particles (Fe) or mixtures of iron-platinum (Fe-Pt) and iron-titanium (Fe-Ti) acting as catalysts. Viability, growth, adhesion, and gene expression of L-929 and retinal precursor (R28) cells were analyzed after nondirect and direct contact.* Results.* Nondirect contact had almost no influence on cell growth, as measured in comparison to reference materials with defined levels of cytotoxicity. Both cell types exhibited good proliferation properties on each MWCNT-coated wafer. Viability ranged from 95.9 to 99.8%, in which better survival was observed for nonfunctionalized MWCNT generated with the Fe-Pt and Fe-Ti catalyst mixtures. R28 cells grown on the MWCNT-coated wafers showed a decreased gene expression associated with neural and glial properties. Expression of the cell cycle-related genes* CCNC*,* MYC*, and* TP53* was slightly downregulated. Cultivation on plasma-treated MWCNT did not lead to additional changes.* Conclusions.* All tested MWCNT-covered slices showed good biocompatibility profiles, confirming that this nanotechnology is a promising tool to improve prostheses bearing electrodes which connect with retinal tissue.

## 1. Introduction

In certain diseases of the neural system, the loss of function can be treated by means of electrical stimulation provided by electronic implants. For example, the cochlear implant has developed to a well established auditory prosthesis used to treat children born deaf or adults with profound hearing loss [1]. The electrodes are located on a linear array that is inserted into the cochlea. After stimulation of the distantly located ganglion neurons, signals are transmitted to the cortex and interpreted as auditory perception.

In the visual system, inherited retinal degenerations, such as retinitis pigmentosa (RP), may lead to blindness and are currently not treatable. RP is characterized by a progressive rod-predominant photoreceptor cell death and affects 0.025% of the population [2]. Its hereditary forms are based on autosomal dominant, autosomal recessive, or X-linked mutations in a variety of genes that are involved, for example, in ciliary trafficking and phototransduction [3, 4]. In order to replace the lost visual input usually mediated by photoreceptor-based transduction into the neural network of the retina, implants were developed to induce visual percepts by electrical stimulation of retinal neurons. These systems, usually referred to as retinal implants (RI), consist of an array of small electrodes, which are either implanted into the subretinal space [5, 6], the intrascleral space [7, 8], or at the epiretinal site [9–11], as well as electronic components to generate stimulation pulses and to manage data and energy transfer.

In contrast to the millions of rods and cones in the healthy retina, RI are equipped with a comparatively low number of electrodes [6, 7, 9]. The EPIRET3 prosthesis, designed and fabricated as a remotely controlled wireless device, includes a microelectrode array consisting of 25 electrodes [11]. Further studies, which were based on the EPIRET3 implant, led to the development of a large flexible multielectrode array [12] as well as flexible microelectrode arrays with an integrated CMOS-chip that will be connected via bus system [13]. These improvements, which result in an increased number and density of stimulating electrodes, but also a suitable three-dimensional design of the electrode itself, narrowing its electrical field, would enhance the prostheses' spatial resolution. The design of such an array has to take several aspects into account. In order to achieve a very high number of electrodes in spite of the limited space for implants within the eye, electrodes have to be closely packed together with low spacing in between and a single electrode has to be small in particular geometrical diameter. However, a small electrode surface is accompanied with an increase in current and charge density, leading to impaired properties of the electrode, and to tissue damage as a consequence of electrochemical reactions [14–17]. Thus, high density electrode arrays are needed, consisting of newly designed electrodes with a small geometric diameter but an enlarged surface area in order to improve tissue biocompatibility and cell adhesion. One promising approach that allows for an extended surface area, while keeping the diameter as small as possible, would be the use of vertically aligned MWCNT-coated on the electrode substrate. Functionalized microelectrodes with integrated aligned MWCNT have been demonstrated to provide an enhanced charge injection limit without heterogeneous charge-transfer reactions that can occur at the electrode surface [18, 19]. Therefore, MWCNT exhibit a promising nanomaterial suitable for neural prostheses. In particular, MWCNT-coated substrates have been shown to support neurite outgrowth, increased neuronal electrical signaling, and synaptic strength [20–22].

In our study, we investigated several vertically aligned MWCNT-coated structures, which differed in the use of distinct Fe catalysts during synthesis. Since oxygen-plasma treatment was described to render the CNT surface hydrophilic [23] and hydrophilic surfaces promote cell growth [24], plasma-treated MWCNT were analyzed in comparison to nonfunctionalized MWCNT. Corresponding to the standard ISO 10993 “biological evaluation of medicinal devices,” part 5 (tests for* in vitro* cytotoxicity) and part 12 (sample preparation and reference materials), biocompatibility was assessed by analyzing the cell growth after direct contact to the vertically aligned MWCNT and after nondirect contact, where the cells were incubated with the respective extractive media. As prescribed by the standard specification, experiments were carried out using L-929 cells, which were reported to be a sensitive cell line [25]. With reference to the surviving cells of the neurosensory retina being the target tissue, growth properties of R28 cells, a retinal precursor cell line that shows both neuronal, and retinal mRNA characteristics [26] were additionally evaluated.

## 2. Materials and Methods

### 2.1. Cell Culture

L-929 cells (ATCC No. CCL-1) were maintained in minimum essential medium (MEM) with Earle's salts (Biochrom, Berlin, Germany) supplemented with 10% fetal calf serum (FCS; PAA Laboratories, Pasching, Austria), 2 mM L-glutamine (Biochrom), 80 U/mL penicillin, and 80 *μ*g/mL streptomycin (Lonza, Basel, Switzerland) at 37°C in a humidified atmosphere of 95% air and 5% CO_2_. The retinal precursor cell line R28, developed by immortalization of postnatal day 6 rat retina [27], was maintained in Dulbecco's modified Eagle's medium (DMEM; Biochrom) supplemented with 10% fetal bovine serum (FBS; PAA Laboratories), 5.5 mL of 100x MEM vitamins and nonessential amino acids (Biochrom), 80 U/mL penicillin, and 80 *μ*g/mL streptomycin at 37°C in a humidified atmosphere of 95% air and 5% CO_2_. Medium was changed 3 times a week. Cells were passaged once or twice a week at a ratio of 1 : 20.

### 2.2. Synthesis of Nonfunctionalized Vertically Aligned MWCNT

**Figure 1:**
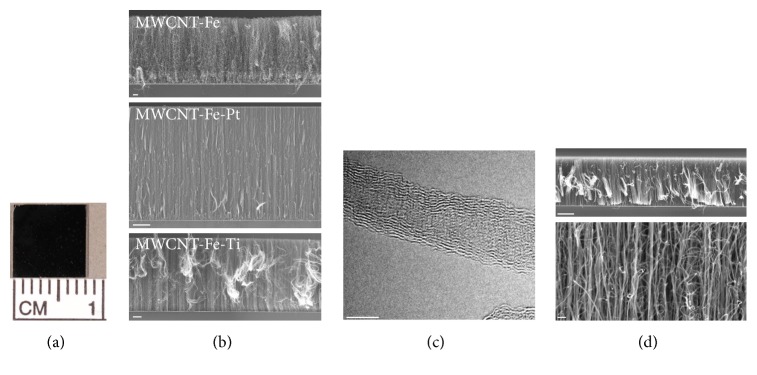
Characterization of vertically aligned MWCNT growth achieved by a nanoscale catalyst layer of Fe, Fe-Pt, and Fe-Ti. (a) Synthesis was carried out on 8 × 8 mm^2^ silicon wafer pieces by chemical vapor deposition. (b) Scanning electron microscopy demonstrated the aligned growth of the synthesized MWCNT with a length of 18 *μ*m for the Fe catalyst (scale bar: 1 *μ*m), 60 *μ*m for the Fe-Pt catalyst (scale bar: 10 *μ*m), and 85 *μ*m for the Fe-Ti catalyst (scale bar: 10 *μ*m). (c) High-resolution transmission electron microscopy demonstrated the integrity and consistency of the Fe-Ti catalyzed MWCNT growth with an outer diameter of 9 nm and an inner diameter of 3 nm (scale bar: 5 nm). (d) Steam sterilization at 121°C for 30 minutes showed no impairment (scale bar upper panel: 10 *μ*m, scale bar lower panel: 100 nm).

Synthesis was carried out on 8 × 8 mm^2^ silicon wafer pieces (Figure 1(a)) by chemical vapor deposition (CVD). Length and homogeneity were improved by an interlayer of 15 nm Al_2_O_3_. Vertically aligned MWCNT growth was achieved by nanoscale layers of Fe as well as mixtures of Fe-Pt and Fe-Ti (thickness of 3 nm) acting as catalysts (Figure 1(b)). Synthesis was carried out at atmospheric pressure and a temperature of 700°C using a 3 : 1 gas mixture of hydrogen and acetylene. Argon was used as feeding gas to achieve gas exchange, needed for MWCNT growth. After a deposition time of 20 min, vertically aligned MWCNT with 18 *μ*m length and 16 nm outer diameters were received by synthesis with pure Fe catalyst. In case of Fe-Pt catalyst, vertical aligned MWCNT with 60 *μ*m length and 28 nm outer diameters were synthesized, whereas Fe-Ti catalyst allowed vertical aligned MWCNT production with 85 *μ*m length and 9 nm outer diameters, as characterized by high-resolution transmission electron microscopy (Figure 1(c)). Prior to the use in cell culture, the wafers had to be sterilized by autoclaving at 121°C for 30 min, which did not affect the MWCNT coating and structure (Figure 1(d)).

### 2.3. Synthesis of Plasma-Treated Vertically Aligned MWCNT

Synthesis was carried out on 8 × 8 mm^2^ silicon wafer pieces by CVD. The interlayer system consisted of undoped silica glass followed by 20 nm titanium and 40 nm titanium nitride on top. A mixture of Fe-Pt with a thickness of 3 nm was used as catalyst. MWCNT synthesis was carried out at atmospheric pressure and a temperature of 620°C using a 3 : 1 gas mixture of hydrogen and acetylene with argon as feeding gas. The deposition time of 40 minutes resulted in MWCNT with 8.6 *μ*m length. Some of the substrates were plasma-treated using a RF plasma at 13.56 MHz with 0.2 W/cm^2^ at a total pressure of 2 mbar with a 1 : 1 gas mixture of oxygen and argon. The treatment time was 30 seconds. Prior to use in cell culture, wafers were sterilized by autoclaving at 121°C for 30 min, which did not affect the MWCNT coating and structure.

### 2.4. Nondirect Contact with MWCNT-Coated Silicon Wafers

According to the description in part 12 of the standard ISO 10993, the materials were incubated at 37°C for 72 h in a humidified atmosphere of 95% air and 5% CO_2_. To avoid blank surfaces, incubation was carried out in one well of a 4-well Nunc*™* Lab-Tek*™* Chamber Slide System (Thermo Fisher Scientific, Waltham, MA) using 0.5 mL of cell culture medium, which was declared as the lowest applicable quantity of liquid. Subsequently, the extractive media were collected and directly applied at different dilutions (1 : 1, 1 : 2, and 1 : 4) to precultivated L-929 and R28 cells (seeded at a density of 31,250 cells/cm^2^ and incubated at 37°C for 24 h). Cell densities were chosen with regard to the subsequent assay to give evaluable results. After another 24 hours of incubation at 37°C, cell viability was assessed using the CellTiter-Glo® Luminescent Cell Viability Assay (Promega, Madison, WI) according to the manufacturer's protocol. The assay is based on quantitation of the adenosine triphosphate, which signals the presence of metabolically active cells. Corresponding to part 12 of the standard ISO 10993, certified positive and negative reference materials (RM; Hatano Research Institute, Hadano, Japan) were used as controls: the positive RM A shows a moderate level of cytotoxicity and consists of a polyurethane film containing 0.1% zinc diethyldithiocarbamate; RM B contains 0.25% zinc dibutyldithiocarbamate and exhibits a weaker cytotoxicity; the negative RM C consists of a high density polyethylene film. In addition, medium incubated without any material was prepared (glass). The reference materials were 8 × 8 mm^2^ in size, analogous to the MWCNT-coated silicon wafers. Each single sample was measured in triplicate; the resulting mean value was used for further analysis. Data (mean ± standard deviation (SD)) were compared to the glass control and analyzed using unpaired two-tailed *t* test.

### 2.5. Direct Contact with MWCNT-Coated Silicon Wafers

L-929 and R28 cells were plated on the wafer slices or on glass at a density of 31,250 cells/cm^2^ and 10,000 cells/cm^2^, respectively. Cell densities were chosen with regard to the subsequent assay to give evaluable results. After a cultivation time of 72 hours at 37°C, cell viability was assessed using 5 *μ*g/mL fluorescein diacetate (FDA, Sigma-Aldrich Chemie, Taufkirchen, Germany) and 10 *μ*g/mL propidium iodide (PI, Sigma-Aldrich Chemie) in PBS/0.1% acetone, which stains live cells green and dead cells red. Immediately after staining, structures were evaluated by fluorescence microscopy in several randomly selected microscopic fields. The total cell numbers were normalized to the respective glass control. The amount of dead cells was calculated as percentage of the corresponding total cell number. Data (mean ± SD) were compared to the glass control and analyzed using unpaired two-tailed *t* test.

For scanning electron microscopy (SEM), R28 cells were plated at a density of 10,000 cells/cm^2^ and incubated at 37°C for 72 hours. After fixation in 3% glutaraldehyde for 1 hour at room temperature and 4 hours at 4°C, cells were washed with 0.1 M Sorensen's phosphate buffer for 15 min, followed by dehydration using an increasing series of ethanol concentrations. The samples were coated with 12.5 nm gold-palladium (Leica EM SCD500; Leica Microsystems, Wetzlar, Germany) and analyzed by SEM (FEI/Philips XL30 ESEM-FEG).

### 2.6. Quantitative Real-Time Polymerase Chain Reaction (qRT-PCR)

Expression levels of genes involved in the cell cycle and representing retinal and neuronal/glial markers were analyzed by qRT-PCR. The primer sequences are detailed in Table 1. R28 cells were plated on the wafer slices or on glass at a density of 10,000 cells/cm^2^. After a cultivation time of 72 hours at 37°C, total RNA was isolated using the RNeasy Mini Kit together with the RNase-free DNase Set (Qiagen, Hilden, Germany) according to the protocol of the manufacturer. Reverse transcription was carried out on 20 ng total RNA using the Reverse Transcription System (Promega). Real-time PCR reactions were performed on a LightCycler 1.2 Instrument using the LightCycler FastStart DNA Master SYBR Green I kit (Roche Diagnostics, Mannheim, Germany) according to the manufacturer's recommendations. For quantification of mRNA expression levels, cDNA samples were run in duplicate or triplicate together with the internal control genes* GAPDH* and* HPRT1*. Reactions were performed with diluted cDNA, corresponding to 0.4 ng of initially used total RNA, and a primer concentration of 0.1 *μ*M and 0.25 *μ*M, respectively. Thermal cycler conditions were set as follows: initial denaturation at 95°C for 10 minutes followed by 50 cycles with denaturation at 95°C for 10 seconds, annealing at 60°C for 8 seconds, and elongation at 72°C for 15 seconds. Melting curve analysis confirmed amplification specificity of each primer pair. Data were processed by LightCycler software 3.5.3 (Roche Diagnostics) and analyzed using the comparative CT (2^−ΔΔCT^) method, which describes relative gene expression [28]. Statistical analysis was performed using one sample two-tailed *t* test.

Even though analysis of the data revealed a constant expression for both internal control genes, the lowest standard deviation was achieved with* GAPDH*. Thus, gene expression levels of all target genes were normalized to the* GAPDH* expression level.

## 3. Results

### 3.1. Effects of Extractive Media on Cell Survival

**Figure 2:**
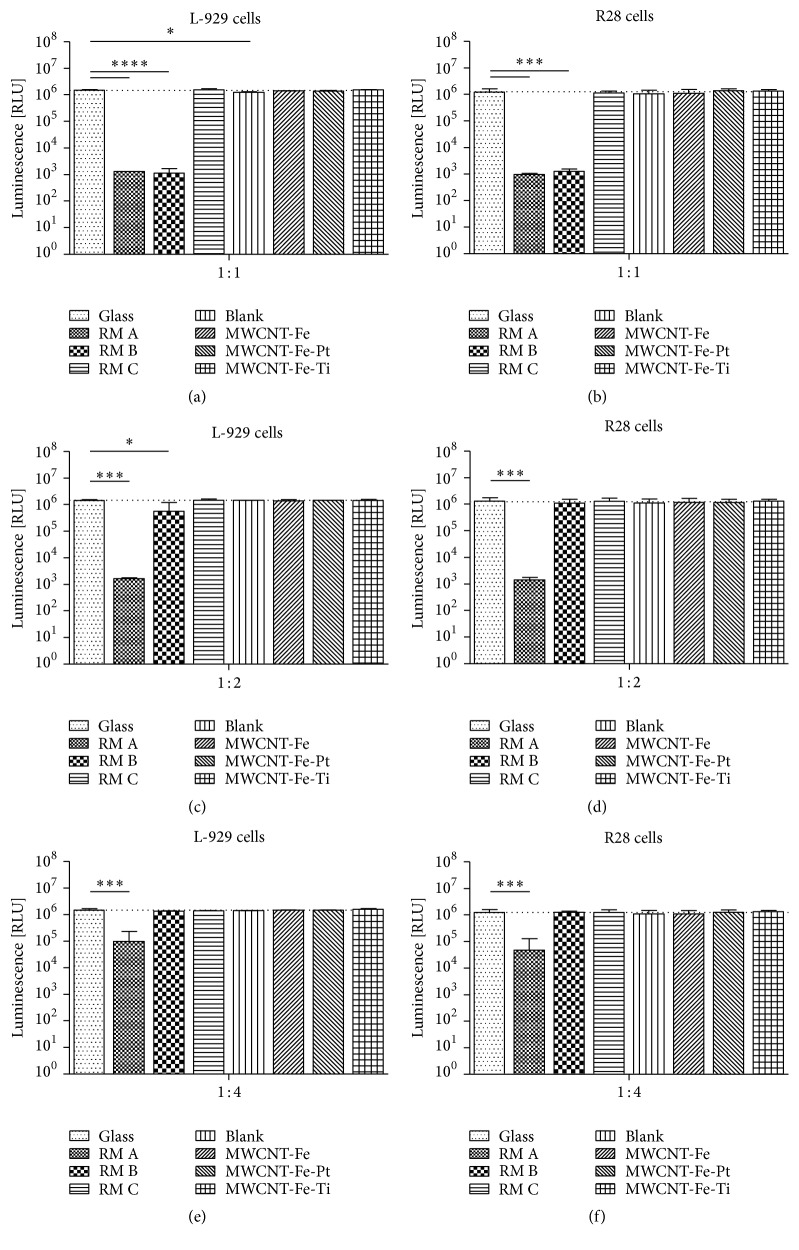
Effects of extractive media on cell survival. Analysis of cell viability in cultures of L-929 cells (*n* = 2 individual experiments) and R28 cells (*n* = 4 individual experiments) incubated for 24 hours with increasing serial dilutions of extractive media obtained from different wafer slices (blank, MWCNT-Fe, MWCNT-Fe-Pt, and MWCNT-Fe-Ti) and certified positive (RM A, RM B) and negative (RM C) reference materials. Extractive medium without the incubation of any material (glass) was used as internal negative control; its mean values are marked by the dotted lines. Data are represented as mean ± SD. They were compared to the glass control and analyzed using unpaired two-tailed *t* test. (a) ^*∗∗∗∗*^ *P* < 0.0001, RM A and RM B; ^*∗*^ *P* = 0.0243, blank. (b) ^*∗∗∗*^ *P* = 0.0010, RM A and RM B. (c) ^*∗∗∗*^ *P* = 0.0002, RM A; ^*∗*^ *P* = 0.0476, RM B. (d) ^*∗∗∗*^ *P* = 0.0009, RM A. (e) ^*∗∗∗*^ *P* = 0.0007, RM A. (f) ^*∗∗∗*^ *P* = 0.0010, RM A.

For both L-929 and R28 cells, incubation with the nontoxic extractive media generated either from the glass substrate or the RM C control exhibited a consistent luminescent output at each applied dilution (Figure 2). Incubation with the nondiluted extractive media generated from the positive RM A and RM B caused a significant reduction of at least 99.9% in the luminescence values (Figures 2(a) and 2(b)), indicating their cytotoxic effects, which in turn decreased with increasing dilutions (Figures 2(c)–2(f)). Direct comparison of RM A and RM B confirmed that RM B exhibited a weaker cytotoxicity than RM A, especially at the higher dilutions 1 : 2 and 1 : 4 (Figures 2(c)–2(f)). The data from extractive media of noncoated (blank) and MWCNT-covered silicon wafers were all in the same range as the glass and the RM C values and thus did not show any evidence of cytotoxicity (Figure 2). One exception was the nondiluted extractive medium of the blank wafer control, which showed a significant decrease of 14.5% in the luminescence values for L-929 cells (Figure 2(a)). Adhesion and coating of any MWCNT on the silicon substrate was neither affected by sterilization nor by incubation in cell culture medium.

### 3.2. Effects of Direct Contact with MWCNT Structures on Cell Viability

**Figure 3:**
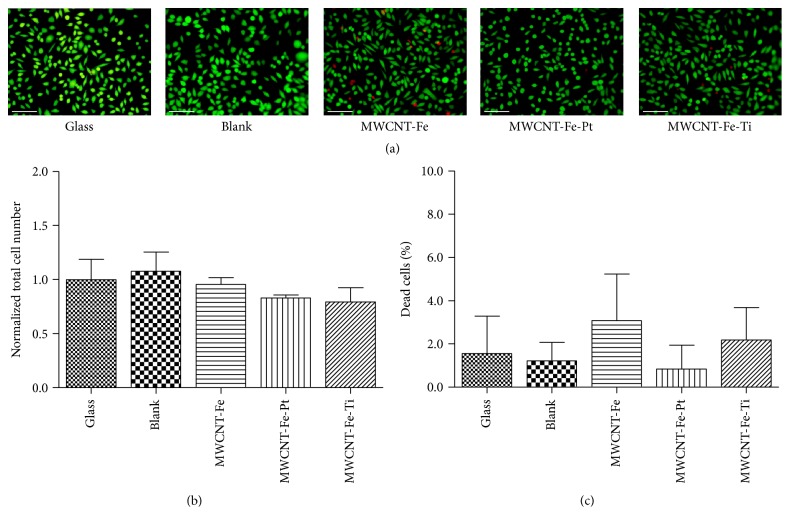
Effects of direct contact on viability of L-929 cells. Cells were plated on a glass substrate and on the blank, MWCNT-Fe, MWCNT-Fe-Pt, and MWCNT-Fe-Ti wafer slices (*n* = 1 experiment), stained using FDA/PI, and counted 72 hours after seeding. (a) Fluorescence microscopy allowed for differentiation between vital (green) and dead (red) cells (scale bar: 100 *μ*m). (b) The total cell numbers were normalized to the respective glass control. (c) The quantity of dead cells is shown as a percentage of the corresponding total cell number. For each substrate, 6 randomly selected microscopic fields were analyzed. Data are represented as mean ± SD. They were compared to the glass control and analyzed using unpaired two-tailed *t* test, showing no significant differences.

L-929 cells were able to grow on the noncoated as well as on the MWCNT-covered silicon wafer slices (Figure 3(a)); the total cell number was comparable between the glass and the blank controls but reduced by 4.4% up to 20.8% in case of the MWCNT-coated structures, with the lowest total cell number determined for the Fe-Ti generated MWCNT (Figure 3(b)). For all structures, the percentage of dead cells was below 6% (Figure 3(c)), with the highest amount observed for the MWCNT-Fe wafer slices (3.1 ± 2.1%), followed by MWCNT-Fe-Ti (2.2 ± 1.5%). The amount of dead cells on MWCNT-Fe-Pt (0.8 ± 1.1%) and the noncoated silicon wafers (1.2 ± 0.9%) was slightly lower than on the glass control (1.5 ± 1.7%).

**Figure 4:**
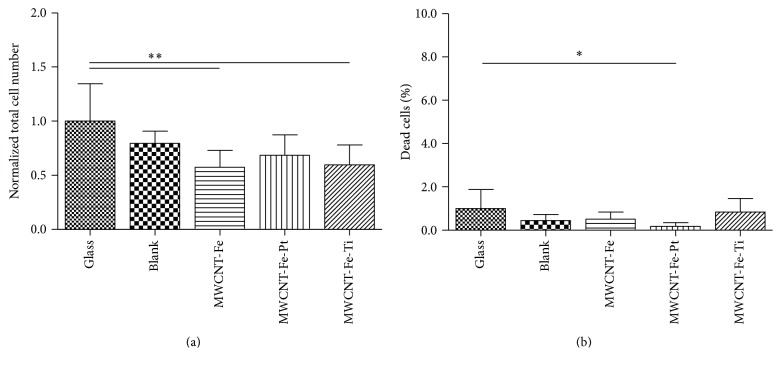
Effects of direct contact on viability of R28 cells. Cells were plated on a glass substrate and on the blank, MWCNT-Fe, MWCNT-Fe-Pt, and MWCNT-Fe-Ti wafer slices (*n* = 2 individual experiments), stained using FDA/PI, and counted 72 hours after seeding. For each substrate, 8 randomly selected microscopic fields were analyzed. Data are represented as mean ± SD. They were compared to the glass control and analyzed using unpaired two-tailed *t* test. (a) The total cell numbers were normalized to the respective glass control. ^*∗∗*^ *P* = 0.0077, MWCNT-Fe; ^*∗∗*^ *P* = 0.0093, MWCNT-Fe-Ti. (b) The quantity of dead cells is shown as a percentage of the corresponding total cell number. ^*∗*^ *P* = 0.0322, MWCNT-Fe.

R28 cells showed the highest total cell number when cultivated on glass (Figure 4(a)). The total cell number on all tested wafer slices was reduced, in some cases significantly, by 20.5% up to 42.6%, with the lowest total cell number detected after cultivation on the MWCNT-Fe-covered and the MWCNT-Fe-Ti-covered silicon wafers (Figure 4(a)). For all tested structures, the percentage of dead cells did not exceed 2% (Figure 4(b)). The highest portion of dead cells was determined on the MWCNT-Fe-Ti substrate (0.8 ± 0.6%), which was comparable to the value evaluated after cultivation on glass (1.0 ± 0.9%).

### 3.3. Gene Expression Profile of R28 Cells after Cultivation on MWCNT Structures

**Figure 5:**
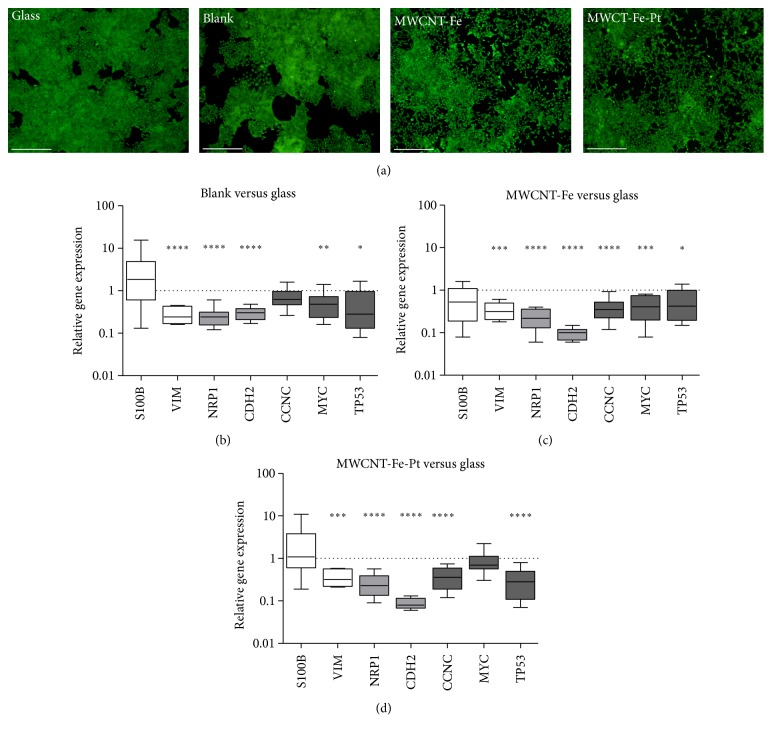
Evaluation of relative gene expression patterns in R28 cells. Cells were plated on a glass substrate and on the blank, MWCNT-Fe, and MWCNT-Fe-Pt wafer slices (*n* = 2 individual experiments). Cultivation was terminated 72 hours after seeding. (a) Fluorescence micrographs showed that R28 cells grown on the MWCNT-Fe and the MWCNT-Fe-Pt wafer slices did not exhibit the characteristic cluster formation as seen on the glass and the blank substrata but showed a lot of separated cells (scale bar: 500 *μ*m). ((b)–(d)) qRT-PCR was performed to analyze the expression of different genes representing neuronal/glial and retinal markers or involved in the cell cycle. Using the comparative CT (2^−ΔΔCT^) method, the relative gene expression ratio of cells cultivated on glass was set to 1. Regarding cultivation on the different wafer slices, values >1 denote upregulation and values <1 denote downregulation of gene expression. Each column represents the median, maximum, minimum, and the 50th percentile of the data. Data were analyzed using one sample two-tailed *t* test. (b) ^*∗∗∗∗*^ *P* < 0.0001,* VIM*,* NRP1*, and* CDH2*; ^*∗∗*^ *P* = 0.0072,* MYC*; ^*∗*^ *P* = 0.0472,* TP53*. (c) ^*∗∗∗*^ *P* = 0.0002,* VIM*; ^*∗∗∗∗*^ *P* < 0.0001,* NRP1*,* CDH2*, and* CCNC*; ^*∗∗∗*^ *P* = 0.0003,* MYC*; ^*∗*^ *P* = 0.0278,* TP53*. (d) ^*∗∗∗*^ *P* = 0.0002,* VIM*; ^*∗∗∗∗*^ *P* < 0.0001,* NRP1*,* CDH2*,* CCNC*, and* TP53*.

In contrast to the cultivation on glass and the noncoated silicon wafers, R28 cells cultivated on MWCNT-Fe and MWCNT-Fe-Pt-covered silicon wafers showed a more separated growth and lacked the morphological characteristic cluster formation (Figure 5(a)). To understand these differences, expression levels of a number of genes essential to the cell cycle and describing neuronal/glial and retinal markers were determined, whereby the values obtained for the cultivation on the wafer pieces were compared to the expression profile evaluated for the cultivation on glass (Figures 5(b)–5(d)).

Compared to the glass control, expression of the retinal cell marker gene* S100B* was not significantly changed in R28 cells cultivated on MWCNT-Fe-Pt silicon wafers (median relative expression ratio: 1.1; Figure 5(d)), while cultivation on noncoated and MWCNT-Fe wafers led to a slight increase (blank: 1.8; Figure 5(b)) and decrease (MWCNT-Fe: 0.5; Figure 5(c)), respectively. The retinal cell marker gene* VIM*, encoding for the intermediate filament protein vimentin, was significantly downregulated when cultivated on the different wafer slices (blank: 0.2; MWCNT-Fe: 0.3; MWCNT-Fe-Pt: 0.3). Its expression was nearly identical to the values observed for the neuronal protein receptor neuropilin (*NRP1*) (blank: 0.2; MWCNT-Fe: 0.2; MWCNT-Fe-Pt: 0.2). Gene expression of the transmembrane protein N-cadherin (*CDH2*) was also significantly downregulated, to an even higher degree in case of the MWCNT-covered silicon wafers (blank: 0.3; MWCNT-Fe: 0.1; MWCNT-Fe-Pt: 0.1). In cells collected after cultivation on MWCNT-covered probes, expression of cyclin C (*CCNC*), a gene that controls nuclear cell division, was decreased to a greater extent than in the noncoated probes (blank: 0.6; MWCNT-Fe: 0.4; MWCNT-Fe-Pt: 0.4). Both the protooncogene* MYC* (blank: 0.5; MWCNT-Fe: 0.4) and the tumor suppressor gene* TP53* (blank: 0.3; MWCNT-Fe: 0.4; MWCNT-Fe-Pt: 0.3) exhibited a significant downregulation in relative gene expression ratio, with exception of the* MYC* expression in cells cultivated on MWCNT-Fe-Pt silicon wafers (median relative expression ratio: 0.7; Figure 5(d)).

### 3.4. Growth Characteristics of R28 Cells after Cultivation on Plasma-Treated MWCNT Structures

**Figure 6:**
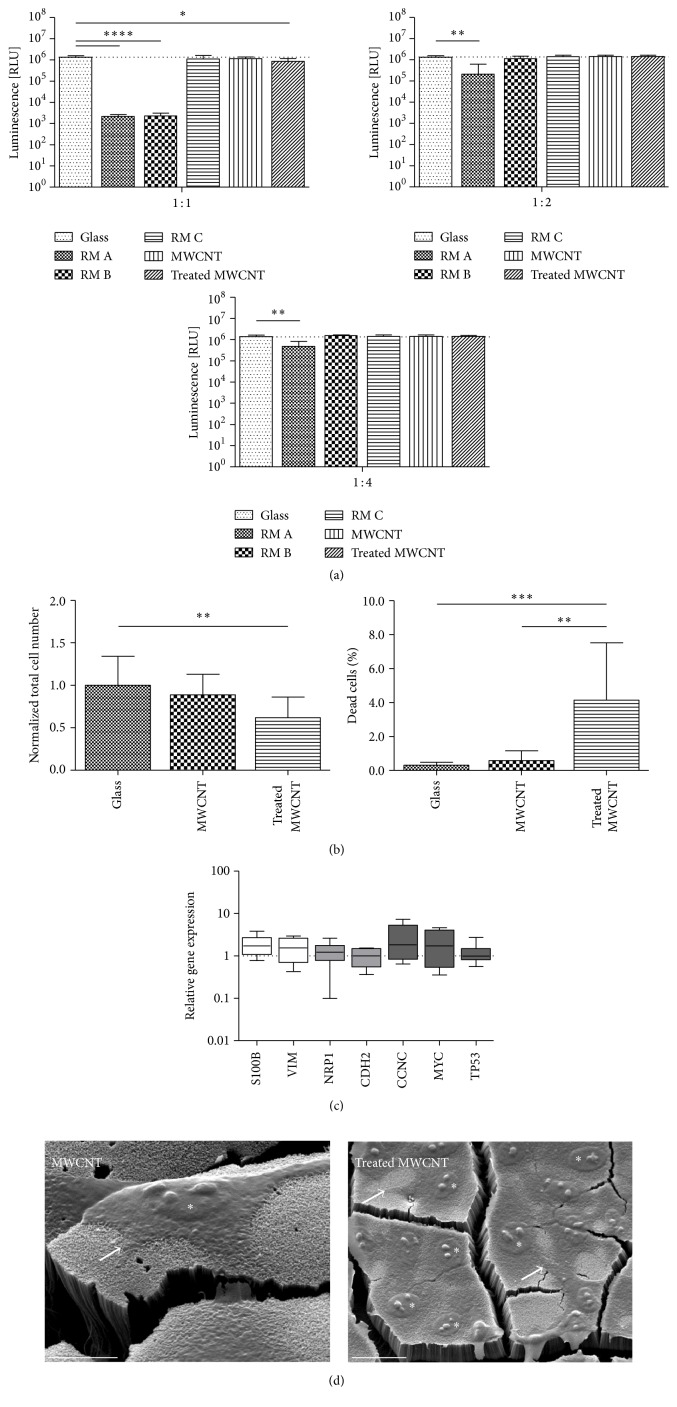
Growth characteristics of R28 cells after cultivation on plasma-treated MWCNT structures. (a) Analysis of cell viability in cultures incubated for 24 hours with increasing serial dilutions of extractive media obtained from nonfunctionalized and plasma-treated MWCNT-Fe-Pt wafer slices and certified RM A, RM B, and RM C (*n* = 4 individual experiments). Extractive medium without the incubation of any material (glass) was used as internal negative control; its mean values are marked by the dotted lines. Data are represented as mean ± SD. They were compared to the glass control and analyzed using unpaired two-tailed *t* test. ^*∗∗∗∗*^ *P* < 0.0001, RM A (1 : 1) and RM B (1 : 1); ^*∗*^ *P* = 0.0319, treated MWCNT (1 : 1); ^*∗∗*^ *P* = 0.0037, RM A (1 : 2); ^*∗∗*^ *P* = 0.0047, RM A (1 : 4). (b) Effects of direct contact on viability of cells grown on glass and on nonfunctionalized and plasma-treated MWCNT-Fe-Pt wafer slices (*n* = 3 individual experiments). Cells were stained using FDA/PI and counted 72 hours after seeding. For each substrate, 12 randomly selected microscopic fields were analyzed. Data are represented as mean ± SD. The total cell numbers were normalized and compared to the glass control and analyzed using unpaired two-tailed *t* test (^*∗∗*^ *P* = 0.0048, treated MWCNT). The quantity of dead cells is shown as a percentage of the corresponding total cell number. They were compared to the treated MWCNT probe and analyzed using unpaired two-tailed *t* test (^*∗∗∗*^ *P* = 0.0007, glass; ^*∗∗*^ *P* = 0.0016, MWCNT). (c) qRT-PCR was used to analyze the expression of different genes representing neuronal/glial and retinal markers or involved in the cell cycle (*n* = 3 individual experiments). Using the comparative CT (2^−ΔΔCT^) method, the relative gene expression ratio of cells cultivated on nonfunctionalized MWCNT was set to 1. For plasma-treated MWCNT, values >1 denote an upregulated and values <1 denote a downregulated gene expression. Each column represents the median, maximum, minimum, and the 50th percentile of the data. Data were analyzed using one sample two-tailed *t* test, showing no significant differences. (d) Scanning electron microscopy images of R28 cells plated on nonfunctionalized (scale bar: 10 *μ*m) and plasma-treated (scale bar: 20 *μ*m) MWCNT-Fe-Pt silicon wafers. Cultivation was terminated 72 hours after seeding. Arrows indicate cell adhesion and individual cell nuclei are marked with stars. Note that the cracks within the MWCNT structures were due to the fixation and drying processes.

Cell viability and the gene expression profile on plasma-treated MWCNT-Fe-Pt were compared to nonfunctionalized MWCNT-Fe-Pt-covered silicon wafers from the same production batch. With regard to the indirect contact, almost no significant cytotoxic effect was observed (Figure 6(a)). Only the nondiluted extract of the plasma-treated MWCNT showed a significant decrease of 36.4% in the luminescent output (Figure 6(a), left panel). R28 cells showed the highest total cell number when cultivated on glass, which decreased by 11.2% for the nonfunctionalized MWCNT-Fe-Pt silicon wafers and by 38.2% for the plasma-treated MWCNT-Fe-Pt silicon wafers (Figure 6(b), left panel). Cultivation on plasma-treated MWCNT-Fe-Pt led to a significant increase in the amount of dead cells (4.1 ± 3.4%; Figure 6(b), right panel), when compared to the cultivation on nonfunctionalized MWCNT silicon wafers (0.6 ± 0.6%) and on glass (0.3 ± 0.2%). Regarding gene expression, growth on the plasma-treated MWCNT did not result in any significant changes when compared to the growth on the nonfunctionalized structures (Figure 6(c)). The median relative expression ratios were almost 1.0 for* TP53* and* CDH2*, whereas a slight upregulation was found for the other tested genes (*NRP1*: 1.2;* VIM*: 1.5;* S100B*: 1.7;* MYC*: 1.7;* CCNC*: 1.8). R28 cell growth on the nonfunctionalized and the plasma-treated MWCNT-Fe-Pt-covered silicon wafers was also analyzed by SEM (Figure 6(d)), which allowed for the illustration of good cell adhesion and biocompatibility properties.

## 4. Discussion

Platinum and iridium oxide are the most frequently used electrode materials for the stimulation of neural tissue; they were shown to be biocompatible and to provide charge injection amounts suitable to achieve neural excitation [29–31]. Nevertheless, in terms of chronic stimulation their stability is controversially discussed. Both materials can be used in standardized processes to fabricate patterned microelectrode arrays. However, in order to produce smaller electrodes for high density electrode arrays, the charge injection limit will become a critical factor. Consequently, electrode materials that provide charge injection via a small diameter are needed, thus increasing charge injection density, but without yielding toxic effects within the tissue and without inducing electrochemical damage at the electrode surface. Based on the geometry and the surface area of the electrode as well as its distance towards the tissue to be stimulated, Shannon [32] determined a model for safe levels of electrical stimulation. Subsequently, this empiric model was adjusted by* in vivo* studies of Butterwick et al. [33], who pointed out that for small electrodes, the charge density is not conserved along the strength-duration curve.

Wang et al. [19] showed that vertically aligned MWCNT pillars can be used as microelectrodes for the* in vitro* stimulation of embryonic rat hippocampal neurons; compared to bare platinum, the MWCNT electrodes offered a 5- to 10-fold higher charge injection ability. Even stimulation charge densities up to 40.7 mC/cm^2^ were reported to be safe [34], suggesting vertically aligned MWCNT grown on microelectrode arrays as a good candidate for the use in neural prostheses. In contrast, safe charge injections of 0.05 up to 5.0 mC/cm^2^ were described for pure metal electrodes [29, 35].* In vivo* application of CNT-modified electrodes in a mouse brain revealed a more efficient detection of action potentials [36]. The functionality of a CNT-coated microelectrode array was demonstrated by its chronic* in vivo* implantation into the motor cortex of a cat [37].

The medical application of nanomaterials in implants represents a contentious issue, as their mechanisms of toxicity may differ from microscale particles or larger devices. MWCNT, whose toxicity depend on their physicochemical properties including structure, surface topology, and manufacturing method, can induce membrane and DNA damage as well as changes in cellular processes and metabolic pathways [38, 39]. However, it is important to distinguish between MWCNT applied as coating on a substrate and their dispersion in a liquid environment. Gladwin et al. [40] showed that medium conditioned with MWCNT was not significantly toxic, whereas MWCNT dispersed in cell culture medium led to a reduction in cell viability. Concerning our final intention of coating a stable MWCNT layer on an electrode array, we only investigated the cytotoxic effects of MWCNT conditioned medium. Additionally, as implanted MWCNT-covered microelectrode structures would get in contact with the remaining cells of the neural tissue of patients, we seeded cells directly onto the different MWCNT-coated silicon wafers. Neither the extractive media nor the direct cell contact caused a significant decrease in cell viability, indicating that no cytotoxic substance was released during incubation of the MWCNT-covered wafer slices in a liquid environment. The ratio of dead cells did not exceed 2% for R28 cells and 6% for L-929 cells. According to part 5 of the standard ISO 10993, a reduction in cell viability of more than 30% is defined as cytotoxic.

Li et al. [41] published a microRNA expression-based method to assess the cytotoxic effect of nanomaterials. In NIH/3T3 cell culture experiments, they showed that MWCNT had less effect on cell survival and cellular morphology than the simultaneous tested nanoparticles and quantum dots, which both were much smaller in size. MWCNT were also found to affect regulation of the NIH/3T3 fibroblast actin cytoskeleton, which corresponded to previously published data that described the effects of carbon nanotubes on human aortic endothelial cells [42]. However, the use of carbon nanotubes together with poly(ethylenedioxythiophene) (PEDOT) for the coating of platinum electrodes implanted into the rat brain exhibited a positive effect compared to bare platinum electrodes in terms of histological changes with less inflammation surrounding the electrodes [43].

Our cell culture experiments revealed that after direct contact with the respective MWCNT, R28 cells showed a more separated growth with a morphologically reduced cluster formation, whereas the growth pattern of L-929 cells was not affected. Accordingly, the decrease of the total cell number was more apparent in cultures of R28 cells. However, this reduced cell survival ratio was in contrast to the results of Tilton et al. [44], who postulated a MWCNT-dependent regulation of pathways that increase cell proliferation. In turn, Bobrinetskii et al. [45] showed a decreased proliferative activity of human embryo fibroblast and glioblastoma cells cultivated on various types of carbon nanotubes. R28 gene expression analysis revealed an increased downregulation in* CDH2* expression after cultivation on MWCNT-covered silicon wafer slices, which may be an explanation for the reduced cell growth.* CDH2* encodes a calcium-dependent cell adhesion molecule and plays an important role in the formation and maturation of synapses in immature neurons [46]. Additionally, expression of* CCNC* and* MYC* was slightly decreased in R28 cells cultured on noncoated and MWCNT-coated silicon wafers, which was consistent with the findings of Liu et al. [47], who described a functional cooperation between* CCNC* and* MYC* to induce cell proliferation.

The more separated R28 cell growth may also be explained by the rather hydrophobic property of the MWCNT surface. In fact, we observed slight water repellence effects when the MWCNT-coated wafer slices were incubated in cell culture medium and suggested that a downstream plasma treatment in an oxygen-argon atmosphere could increase the hydrophilicity of the MWCNT surface and thus improve cell adhesion and cell growth. However, compared to nonfunctionalized MWCNT structures, gene expression was almost not affected, whereas the total cell number significantly declined and the percentage of dead cells significantly increased. Nevertheless, more than 95% of the cells were vital.

Our study demonstrated that the analyzed MWCNT fabricated with different catalytic agents were biocompatible for L-929 cells as an ISO 10993 standard biological test system. Although the L-929 cell line is described to be sensitive for cytotoxicity testing, its fibroblastic morphology does not reflect the properties of the retina, which is the target tissue that will ultimately connect with a retinal implant-embedded MWCNT-coated electrode array. Beside the loss of the sensory retina, retinal degenerations are characterized by a downstream remodeling of the neural retina, where Müller cells increase the synthesis of intermediate filaments, forming a dense layer in the subretinal space [48]. R28 cells are known to express the glial marker genes* S100B* and* VIM* [49], whose expression is ubiquitously found in Müller cells [50, 51]. Regarding this, R28 cells are in better agreement with the genetic characteristics of a remodeled neural retina. The results showed that the tested MWCNT were biocompatible for R28 cells, albeit to a slightly reduced extent, because of minor changes in the growth pattern and gene expression.

However, the cell lines only give a first indication of the biocompatibility of the various MWCNT themselves and allow the assumption that implantation of a MWCNT-coated microelectrode array would not lead to immunogenic rejections. The production of MWCNT-functionalized microelectrode arrays is a matter of ongoing research. Future experiments will comprise organotypic cultures of retinas with induced photoreceptor degeneration [52, 53] as well as the* in vivo* implantation in an animal model of retinal degeneration. The studies will also involve the biocompatibility testing of MWCNT-coated microelectrode arrays after application of stimulation pulses, in order to exclude a possible leaching of the Fe, Fe-Pt, or Fe-Ti catalysts.

Despite the negligible differences in the biocompatibility, which were not as evident as one would expect from cytotoxic materials, MWCNT-covered microelectrode arrays provide a promising approach to electrically stimulate remaining neural cells in patients with neurodegenerative diseases.

## 5. Conclusions

Different Fe catalyst mixtures were tested to improve the synthesis of vertically aligned MWCNT on silicon wafers. All tested MWCNT-covered slices showed good biocompatibility profiles; as for the direct contact, the amount of dead cells was only one-fifth to one-fifteenth of those what is defined as cytotoxic. Thus, this technology is considered as a promising tool to further improve microelectrode array systems developed to connect with remaining retinal cells. Our results justify continuing the biocompatibility and efficacy testing* in vivo* in a rabbit model, where the surgical techniques and following treatments are well established in our department.

## Figures and Tables

**Table 1:** Primers used in qRT-PCR.

		Sequence (5′ to 3′)
*GAPDH*	Upstream	tgg gaa gct ggt cat caa c
	Downstream	gca tca ccc cat ttg atg tt

*HPRT1*	Upstream	ctc ctc aga ccg ctt ttc c
	Downstream	tca taa cct ggt tca tca tca cta a

*S100B*	Upstream	aag gga gtt ccc tgg gtt t
	Downstream	cac tgg tcc agg tct ttc att

*VIM*	Upstream	aac act cct gat taa gac ggt tg
	Downstream	tca tcg tgg tgc tga gaa gt

*NRP1*	Upstream	cat agt ggg ctc gga ctg a
	Downstream	ggt cca gct gta ggc act tc

*CDH2*	Upstream	cca tca tcg cga tac ttc tg
	Downstream	cca tac cac gaa cat gag ga

*CCNC*	Upstream	aaa acc acc tcc gaa cag tg
	Downstream	gat tgg ctg tag cta gag ttc tga c

*MYC*	Upstream	gct cct cgc gtt att tga ag
	Downstream	gca tcg tcg tga ctg tcg

*TP53*	Upstream	aga gag cac tgc cca cca
	Downstream	aac atc tcg aag cgc tca c
